# Supplementary material for: Combining sCD163 with CA 19-9 Increases the Predictiveness of Pancreatic Ductal Adenocarcinoma
Source: Cancers (Basel). 2023 Jan 31;15(3):897. doi: 10.3390/cancers15030897 (PMC9913074; doi:10.3390/cancers15030897)

Supplementary data

Manuscript title: Plasma sCD163 as a biomarker in patients with pancreatic cancer

Julia S. Johansen et al.

Supplementary Table S1: Studies evaluating CD163 in tumor tissue in patients with PDAC.

Supplementary Table S2: Studies evaluating sCD163 in plasma.

Supplementary Table S3: Chemotherapy regiments

Supplementary Table S4: REMARK guidelines checklist

Supplementary Table S5: Pairwise Wilcoxon signed-rank tests

Supplementary Figure S1: Kaplan Meier survival curve

Supplementary Figure S2: Box plot showing distribution of sCD163, separated by gender

Supplementary Table S1 Main characteristics of four studies evaluating the prognostic value of CD163 in tumor tissue in patients with PDAC.

Author	Year	Analysis and findings	HR (95% CI)	p-value	Comment
Shi B[10]	2021	The prognostic value of CD163 infiltration was evaluated using cox proportional hazards regression model. CD163 was determined as an independent prognostic marker associated with inferior OS.	1.53 (1.06, 2.12)	0.007	CD163 infiltration was evaluated using paraffin-embedded tissues incubated with monoclonal mouse anti-human CD163 antibody
Knudsen ES[7]	2017	Survival curves were estimated by the Kaplan-Meier method and compared using the two-sided log-rank test. High infiltration of CD163 within the tumor microenvironment was significantly associated with inferior OS.	NR	0.001	CD163 infiltration was evaluated using paraffin-embedded tissues to construct TMAs. The TMAs were then stained with monoclonal mouse anti-human CD163 antibodies
Hutcheson J[9]	2016	Survival curves were estimated by the Kaplan-Meier method and compared using the two-sided log-rank test. High infiltration of CD163 within the tumor microenvironment was significantly associated with inferior OS.	2.06 (NR)	0.005	CD163 infiltration was evaluated using paraffin-embedded tissues to construct TMAs. The TMAs were then stained with monoclonal mouse anti-human CD163 antibodies
Chen SJ[8]	2015	Survival curves were estimated by the Kaplan-Meier method and compared using the two-sided log-rank test. High infiltration of CD163, both centrally and peripherally, within the tumor microenvironment was significantly associated with inferior OS.	NR	CD163 centrally: 0.004<br>CD163 peripherally: 0.001	CD163 infiltration was evaluated using paraffin-embedded tissues incubated with monoclonal mouse anti-human CD163 antibody

Abbreviations: CI, confidence interval; HR, hazard ratio; NR, not reported; OS, overall survival; PDAC, pancreatic ductal adenocarcinoma; TMA, tumor microarray

Supplementary Table S2 Main characteristics of eleven studies evaluating the prognostic value of plasma sCD163.

Author	Year	Type of cancer	Median (range)	HR (95% CI)	p-value	Comment
Davidsson S [25]	2022	Renal cell carcinoma	0.74 (0.62, 0.89)	NR	NR	sCD163 was measured using a sandwich ELISA with monoclonal anti-CD163 antibody.<br>This study chose CSD as the only survival outcome. No association between sCD163 and CSD was found.<br>This study found significantly elevated levels of sCD163 in patients with malignant tumors compared to patients with benign tumors.
Vajavaara H[24]	2021	Diffuse large B-cell lymphoma	Trial cohort:<br>1.16 µg/ml (0.37, 3.62)	5.08 (0.98, 26.39)	0.053	sCD163 was measured using a sandwich ELISA with anti-CD163 rabbit polyclonal antibody (population-based cohort), and with sandwich ELISA Quantikine with monoclonal anti-CD163 antibody (trial cohort). .<br>This study found significantly elevated levels of sCD163 in patients with cancer compared to healthy controls.
			Population-based cohort:<br>2.95 µg/ml (0.87, 3.00)	2.21 (0.99, 4.94)	0.052	
			Healthy controls:<br>0.437 µg/ml (0.220, 0.518)			
Krijgsman D[17]	2020	Colorectal cancer	2.0 µg/ml (NR)	2.2 (1.0, 4.6)	0.040	sCD163 was measured using a sandwich ELISA with anti-CD163 rabbit polyclonal antibody.<br>The association with OS was not significant in multivariate analysis.
Ding D[16]	2017	Gastric cancer	Patients with cancer:<br>0.63 µg/ml (0.29, 1.76)<br>Healthy controls:<br>0.43 µg/ml (0.21, 0.73)	3.76 (1.83, 7.73)	<0.001	sCD163 was measured using a sandwich ELISA with anti-CD163 rabbit polyclonal antibody.<br>The association with OS was not significant in multivariable analysis.
						This study found significantly elevated levels of sCD163 in patients with cancer compared to healthy controls.

Kanakry JA[22]	2016	Classical Hodgkin lymphoma	NR	2.89 (1.65, 5.05)	0.0002	sCD163 was measured using multiplex (Luminex) bead array immunoassays.
Kazankov K[19]	2015	Hepatocellular carcinoma	Patients with cancer: 5.6 µg/ml (3.5, 8.0)<br>Healthy controls: 2.0 µg/ml (1.5, 2.7)	PFS: 1.42 (1.10, 1.82)	0.006	sCD163 was measured using a sandwich ELISA with anti-CD163 rabbit polyclonal antibody.<br>This study found a significant association with PFS. No significant association was found with OS.<br>This study found significantly elevated levels of sCD163 in patients with cancer compared to healthy controls.
Nederby L[23]	2015	B-cell lymphocytic leukemia	2.09 µg/ml (0.77, 9.01)	5.10 (1.20, 28.17)	0.029	sCD163 was measured using a sandwich ELISA with anti-CD163 rabbit polyclonal antibody.<br>This study chose PFS as the only survival outcome.
Andersen MN[15]	2014	Multiple myeloma	2.09 µg/ml (1.52, 2.84)	1.82 (1.15, 2.86)	0.010	sCD163 was measured using a sandwich ELISA with anti-CD163 rabbit polyclonal antibody.
Waidmann O[20]	2013	Hepatocellular carcinoma	NR	0.25 (0.09, 0.71)	0.009	sCD163 was measured using Macro163 sandwich ELISA with anti-CD163 polyclonal antibody.<br>This study reported HR for patients with low sCD163 levels, compared to patients with high sCD163 levels.
No JH[21]	2012	Epithelial ovarian cancer	1.71 µg/ml (1.05, 2.86)	DFS: 3.1 (1.2, 8.1)	0.016	sCD163 was measured using a sandwich ELISA with monoclonal anti-CD163 antibody.<br>This study found a significant association with DFS in a Cox proportional hazards model.<br>A significant association with OS was found (log-rank test (p=0.027)).
Jensen TO[18]	2009	Melanoma	NR	1.4 (1.1, 1.7)	0.01	sCD163 was measured using a sandwich ELISA with anti-CD163 rabbit polyclonal antibody.

Abbreviations: CI, confidence interval; CSD, cancer specific death; DFS, disease free survival; ELISA, enzyme-linked immunosorbent assay; HR, hazard ratio; NR, not reported; OS, overall survival; PFS, progression free survival; sCD163, soluble CD163

Supplementary Table S3 The number of patients with PDAC treated with the different standard types of chemotherapy

Chemotherapy type	Number (%)
**Adjuvant chemotherapy**	74 (29.0%)
Gemicitabine	52 (20.4%)
Gemicitabine + capecitabine	6 (2.4%)
Gemicitabine + nab-paclitaxel	2 (0.8%)
FOLFIRINOX	14 (5.5%)
**Palliative chemotherapy**	145 (56.9)
Gemicitabine	47 (18.4)
Gemicitabine + capecitabine	14 (5.5)
Gemicitabine + nab-paclitaxel	45 (17.7)
FOLFIRINOX	39 (15.3)
**Never received chemotherapy**	36 (14.1)

Abbreviations: FOLFIRINOX, 5-Fluorouracil, irinotecan and oxaliplatin

Supplementary Table S4 REMARK guidelines checklist

	Item to be reported	Page no.
**INTRODUCTION**		
1	State the marker examined, the study objectives, and any pre-specified hypotheses.	4-7
**MATERIALS AND METHODS**		
Patients		
2	Describe the characteristics (e.g., disease stage or co-morbidities) of the study patients, including their source and inclusion and exclusion criteria.	8-9
3	Describe treatments received and how chosen (e.g., randomized or rule-based).	8
Specimen characteristics		
4	Describe type of biological material used (including control samples) and methods of preservation and storage.	10
Assay methods		
5	Specify the assay method used and provide (or reference) a detailed protocol, including specific reagents or kits used, quality control procedures, reproducibility assessments, quantitation methods, and scoring and reporting protocols. Specify whether and how assays were performed blinded to the study endpoint.	10-11
Study design		
6	State the method of case selection, including whether prospective or retrospective and whether stratification or matching (e.g., by stage of disease or age) was used. Specify the time period from which cases were taken, the end of the follow-up period, and the median follow-up time.	8-9
7	Precisely define all clinical endpoints examined.	14
8	List all candidate variables initially examined or considered for inclusion in models.	8-9
9	Give rationale for sample size; if the study was designed to detect a specified effect size, give the target power and effect size.	11
Statistical analysis methods		
10	Specify all statistical methods, including details of any variable selection procedures and other model-building issues, how model assumptions were verified, and how missing data were handled.	11-12
11	Clarify how marker values were handled in the analyses; if relevant, describe methods used for cutpoint determination.	11-12
**RESULTS**		
Data		
12	Describe the flow of patients through the study, including the number of patients included in each stage of the analysis (a diagram may be helpful) and reasons for dropout. Specifically, both overall and for each subgroup extensively examined report the numbers of patients and the number of events.	13
13	Report distributions of basic demographic characteristics (at least age and sex), standard (disease-specific) prognostic variables, and tumor marker, including numbers of missing values.	13
Analysis and presentation		
14	Show the relation of the marker to standard prognostic variables.	13
15	Present univariable analyses showing the relation between the marker and outcome, with the estimated effect (e.g., hazard ratio and survival probability). Preferably provide similar analyses for all other variables being analyzed. For the effect of a tumor marker on a time-to-event outcome, a Kaplan-Meier plot is recommended.	14
16	For key multivariable analyses, report estimated effects (e.g., hazard ratio) with confidence intervals for the marker and, at least for the final model, all other variables in the model.	14
17	Among reported results, provide estimated effects with confidence intervals from an analysis in which the marker and standard prognostic variables are included, regardless of their statistical significance.	14
18	If done, report results of further investigations, such as checking assumptions, sensitivity analyses, and internal validation.	-
**DISCUSSION**		
19	Interpret the results in the context of the pre-specified hypotheses and other relevant studies; include a discussion of limitations of the study.	15-17
20	Discuss implications for future research and clinical value.	17

Supplementary Table S5 Pairwise Wilcoxon signed-rank tests for plasma sCD163 concentrations in patients with PDAC (grouped by cancer stage) and healthy controls. The contents of this table are graphically depicted as a Box plot in Figure 4.

Pairwise comparisons	Group 1 (N)	Group 2 (N)	p-value	Adjusted p-value
	Stage 1 (12)	Stage 2 (48)	0.193	0.965
	Stage 1 (12)	Stage 3 (42)	0.333	1
	Stage 1 (12)	Stage 4 (153)	0.636	1
	Stage 1 (12)	Donors (80)	**0.005**	**0.033**
	Stage 2 (48)	Stage 3 (42)	0.752	1
	Stage 2 (48)	Stage 4 (153)	0.098	0.590
	Stage 2 (48)	Donors (80)	**<0.0001**	**<0.0001**
	Stage 3 (42)	Stage 4 (153)	0.318	1
	Stage 3 (42)	Donors (80)	**<0.0001**	**<0.0001**
	Stage 4 (153)	Donors (80)	**<0.0001**	**<0.0001**

Supplementary Figure S1 Kaplan Meier survival curve showing OS according to stage in patients with PDAC

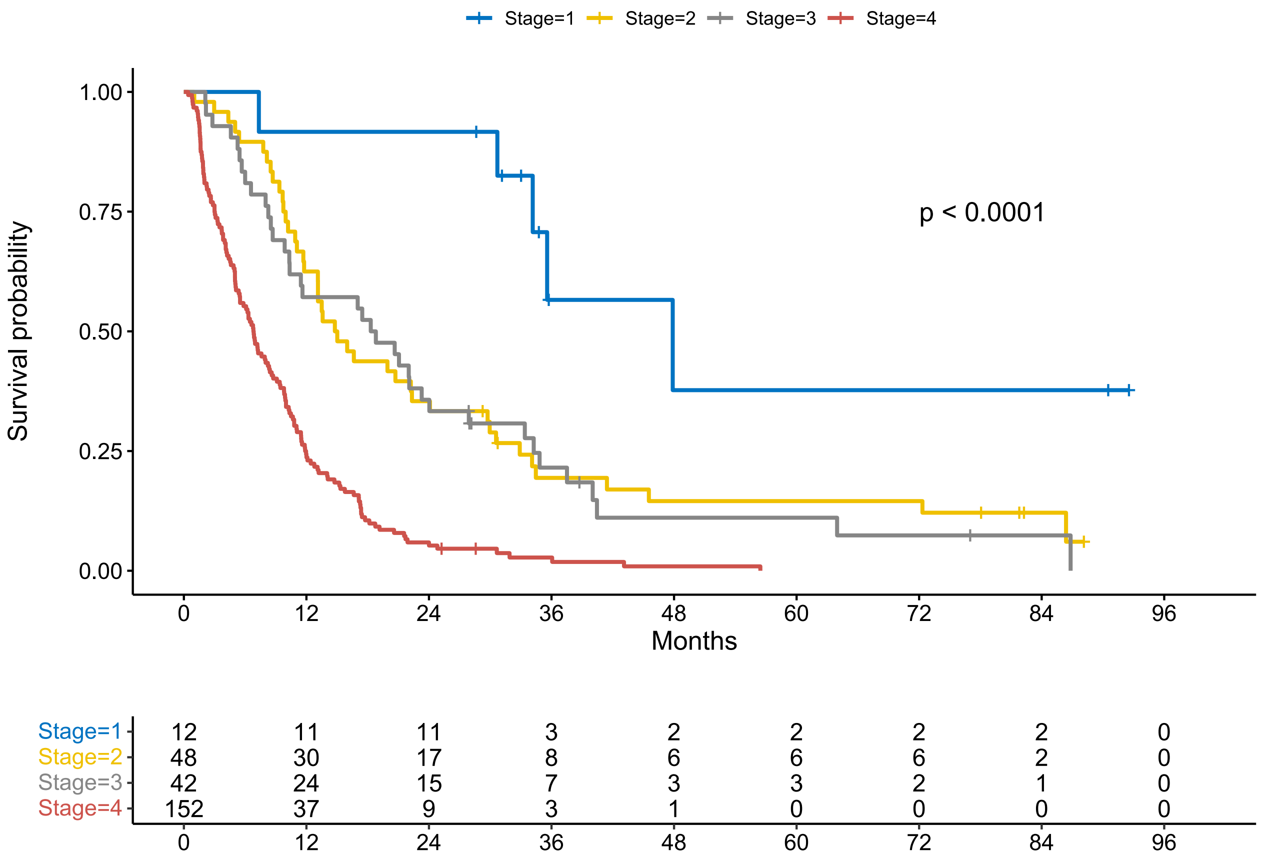

Supplementary Figure S2 Box plot showing the distribution of plasma sCD163 concentrations in patients with PDAC at time of diagnosis, stratified by stage, and in healthy controls, separated by gender.

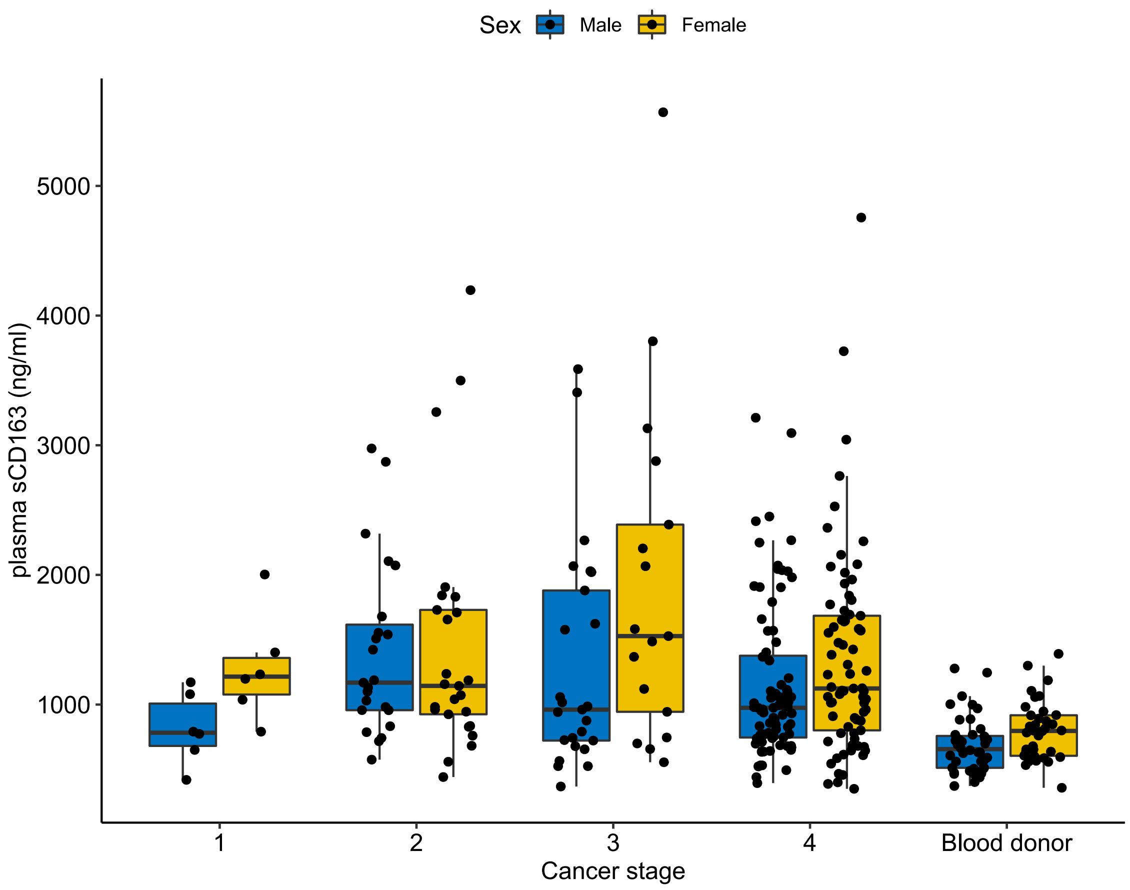

Supplement: Supplementary file 1 [file cancers-15-00897-s001.zip › cancers-2109712-supplementary.pdf]
